# High asymptomatic malaria among seasonal migrant workers departing to home from malaria endemic areas in northwest Ethiopia

**DOI:** 10.1186/s12936-022-04211-9

**Published:** 2022-06-11

**Authors:** Tesfaye Tilaye, Belay Tessema, Kassahun Alemu

**Affiliations:** 1 Institute of Public Health, University of Gondar, Gondar, Ethiopia; 2 Department of Medical Microbiology, College of Medicine and Health Sciences, University of Gondar, Gondar, Ethiopia

**Keywords:** Asymptomatic malaria, Malaria transmission, Seasonal migrant workers

## Abstract

**Background:**

In Ethiopia, thousands of seasonal migrant workers travel from non-malaria or mild malaria transmission areas to malaria-endemic areas for seasonal farm activities. Most of these migrants stay in the farm areas for land preparation, plowing, planting, weeding, and harvesting for a specific period and return to their living areas. However, there is limited evidence of how seasonal migrant workers contribute to the transmission of malaria to new or less malaria transmission areas.

**Methods:**

A cross-sectional study was conducted at the departure phase of seasonal migrant workers in the Metema district from September 2018 to October 2019. A total of 1208 seasonal migrant workers were interviewed during their departure from farm sites to their homes. The face-to-face interviews were performed using a pretested structured questionnaire. Moreover, blood samples were collected from each study participant for microscopic malaria parasite examination. The data were fitted with the logistic regression model to estimate the predictors of malaria transmission.

**Results:**

At departure to home, the prevalence of malaria among seasonal migrant workers was 17.5% (15.6–19.45%). Approximately 71.80% (177/212) of the cases were *Plasmodium falciparum,* and 28.20% (35/212) were *Plasmodium vivax*. Most seasonal migrant workers 934 (77.4%) were from rural residences and highlanders 660 (55%). Most 661 (55.4%) of the migrants visited two and more farm sites during their stay at development corridors for harvesting activities. Approximately 116 (54.7%) asymptomatic malaria cases returned to the Dembia 46 (21.7%), Chilaga 46 (19.8%) and Metema 28 (13.2%) districts.

**Conclusion:**

In this study, asymptomatic malaria remains high among seasonal migrant workers departing to home from malaria endemic areas. This may fuel a resurgence of malaria transmission in the high lands and cause challenges to the country's malaria prevention and elimination efforts. Hence, tailored interventions for seasonal migrant workers could be in place to enhance malaria control and elimination in Ethiopia, such as asymptomatic malaria test and treat positive cases at departure and transit, and integration between malaria officers at their origin and departure for further follow-up to decrease any risk of spread at the origin.

## Background

Malaria is continuing to be a global public health problem [1]. It is caused by *Plasmodium* parasites and, in most cases, transmitted through the bites of female *Anopheles* mosquitoes. Among the five *Plasmodium* parasite species that cause malaria in humans, *Plasmodium falciparum* and *Plasmodium vivax* are widely distributed around the globe. In the World Health Organization (WHO) African region, *P. falciparum* is the most prevalent [2, 3].

Globally, between 2010 and 2018, a significant decline in the malaria incidence rate was documented, from 71 to 57 cases per 1000 population at risk [4]. However, after 2016, the success started slowing down and remained a major burden of disease. In 2018, the World Malaria Report indicated an estimated 228 million malaria cases and 405,000 deaths globally. The WHO African region shared 93% of all cases and 94% of all deaths [5]. In Ethiopia, over 68% of the country’s landmass is still malarial, and 60% of the population is at risk of malaria infection [6–8]. In 2016, an estimated three million new malaria cases and five thousand deaths were reported [9], which showed a 50% malaria incidence and mortality decline compared to the previous years [10]. This achievement was associated with improved coverage of long-lasting insecticidal nets (LLINs), indoor residual spraying (IRS), malaria diagnoses using rapid diagnostic tests (RDTs), prompt treatment using artemisinin-based combination therapy (ACT), and destruction of mosquito breeding sites using environmental management, from 2005 to 2015 [10]. However, malaria remains among the ten leading causes of morbidity and mortality [1, 11]. Moreover, the country has not yet established a robust surveillance and health management information system to monitor mortality and incidence rates of malaria [11].

Migration to countries and within countries is usually cyclical and seasonal [12]. In Asia and Africa, people are moving from country to country or within the country for economic purposes, mostly for agricultural activities [13]. Most agricultural farms are found in high malaria transmission areas, and movement from malaria-free or low malaria areas to these areas puts migrants at risk of malaria infection [14]. This would result in a resurgence of malaria, outbreaks, the spread of malaria parasites and drug-resistant malaria parasites, and challenges in malaria prevention and elimination activities [15, 16]. Studies revealed that the risk of confirmed malaria in high land areas was up to seven times higher in people who had a travel history to high malaria transmission areas than in those who did not [17]. Moreover, studies have identified risk factors that increase exposure to malaria among seasonal migrants, including being a male [18], having a low education status and low knowledge of malaria prevention methods [16, 19, 20], sleeping outside the house, and working at night, low treatment-seeking behaviour [21, 22], and low access to and utilization of insecticide-treated nets (ITNs) [23–25].

Studies have shown that population movement is closely linked to malaria spread, resurgence, and outbreaks [26–28], and countries have found migration to be a key player in the reintroduction of malaria cases [29], posing challenges to the control and elimination of malaria [30, 31]. African countries were particularly affected by unrecognized migrants and were unable to continue with the malaria elimination programme. As a result, following the renewal of the malaria elimination paradigm in recent times, the population movement has received recognition, especially in countries that eliminated malaria and those that are moving to eliminate malaria and sustain malaria elimination [31].

In Ethiopia, most migration is seasonal or cyclical [24]. Seasonal migrant workers are key players either as active transmitters or passive acquirers. As active transmitters, they harbour the parasite due to their low level of immunity or lack of immunity to malaria and are at high risk of malaria infection and transmit the disease to areas of low or sporadic transmission as passive acquirers; they are exposed to the disease through movement from one environment to another [29, 32, 33]. Moreover, it has been shown that seasonal and short-term migrant workers are more at risk of malaria infection and play a central role in malaria transmission due to travelling to endemic areas with no immunity or partial immunity [26]. Health facility-based malaria studies revealed a high prevalence of malaria parasites among returnees from malaria-endemic areas [8, 23]. Therefore, seasonal migrant workers can reintroduce the parasite and initiate a resurgence and an outbreak of malaria when they return to their permanent living home where it might be malaria receptive [31].

The magnitude of malaria in seasonal migrant workers during harvest time and their role in malaria transmission to new or low malaria transmission areas is less known. This study assessed asymptomatic malaria prevalence and associated risk factors among seasonal migrant workers at departure return to home. The outcome of this study could provide valid information and insight that will bridge the knowledge gap for the programmatic improvement of malaria prevention and control in Ethiopia tailored to seasonal migrant workers.

## Methods

### Study area

**Figure 1:**
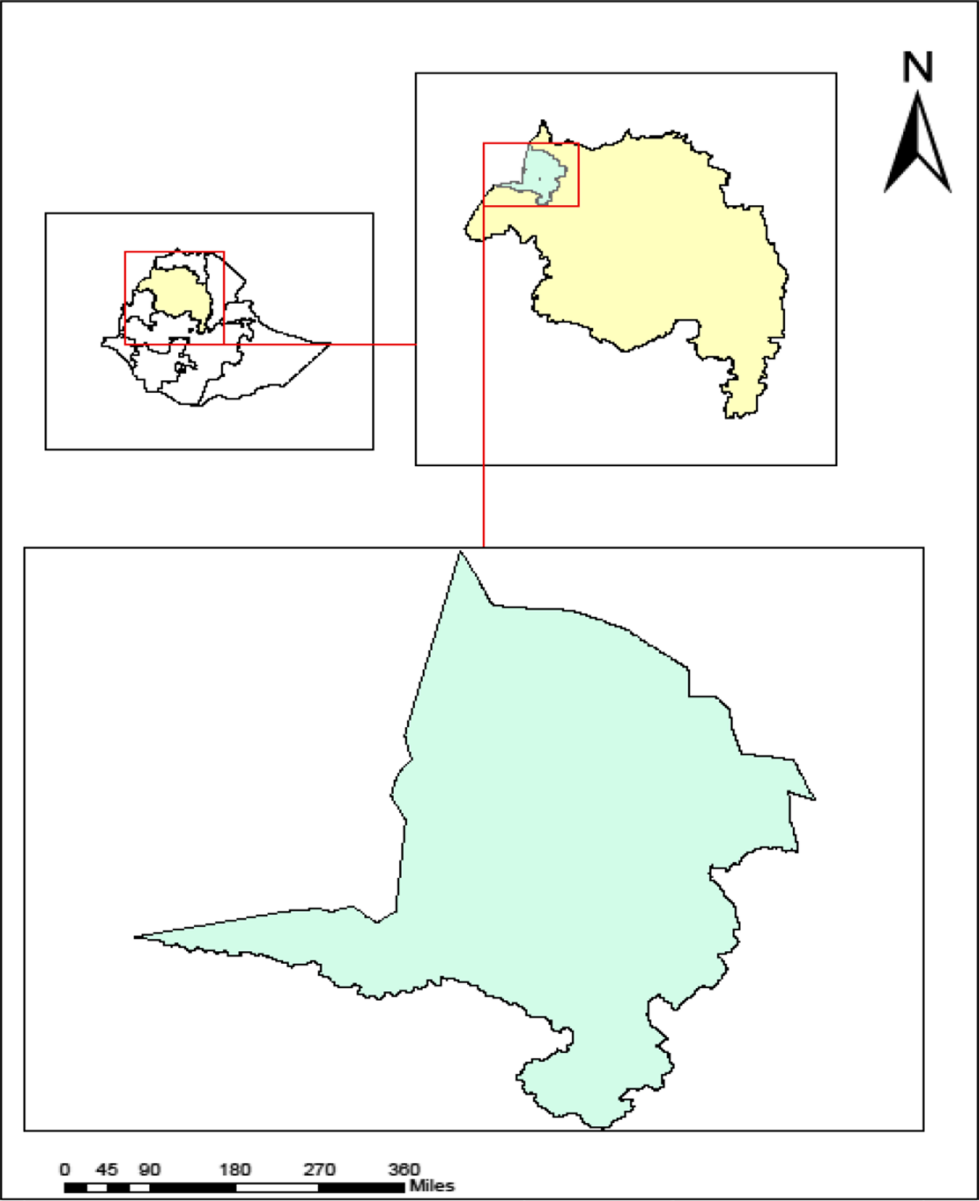
Map of Metema district, Northwest Ethiopia

The study was conducted in the Metema district of northwest Ethiopia (Fig. 1). It is one of the nine agricultural investment districts with a total permanent resident population of 154,618 [34]. The district shares boundaries with three districts, Quara, West Armachio, Chilga, and Sudan. The study area is lowland with an average altitude of 750 m (500–1000 m). The mean annual rainfall for the area ranges from approximately 850–1000 mm. The district has 26 rural and three urban kebeles one district hospital, five health centres, and 26 health posts as well as private facilities: 47 clinics, 5 medium diagnostic laboratories, 14 drug vendors, 9 rural drug shops, and 21 legal traditional medical sectors [35].

Metema district is one of the seven agricultural investment areas receiving an estimated 120,000 seasonal migrant workers mainly from the Amhara region with various climatic zones: highland, midland, and lowland. These migrants are mostly engaged in farmland preparation/farm site clearing, farming, weeding, and harvesting of sesame, sorghum, and cotton products at their destination. Site clearing, farming, planting, and weeding take place from May to the second week of September. Harvesting of sesame occurs from the end of September to December, the major malaria transmission season following the main rainy season (June to September). Then, few migrants will remain at destination from 1 month to 6 months to collect Sorghum and cotton.

### Sample size, sampling, and data collection

A single population proportion was used to calculate the sample size with an estimated proportion of 27.5% [36], an acceptable difference = 3%, α = 5% (95% confidence level), and a 10% nonresponse rate. Therefore, a total sample size of 1256 was determined**.** The response rate was 96% (1208).

Participants were identified at departure just after they completed the contractual agreement and left the farm sites to areas where they stayed for hours to a few days until they got transport to their origin/home. Returnees were gathered in two towns, Delelo one and Delelo two, where they were approached to identify the average flow of seasonal migrant workers to these towns and to identify the time when to get most of the study subjects. Registrations of migrants were performed at the departure sites by data collectors daily, and a random sampling collection method was employed. Data collection at the departure helps to understand the number of malaria parasites being carried by seasonal migrant workers from farm sites to their origin or living home.

Data on sociodemographic characteristics and knowledge of malaria prevention methods were collected from the departure phase for malaria infection using a structured questionnaire. The questionnaire comprised independent predictors such as sociodemographic characteristics (sex, age, education, occupation, religion, ethnicity, and salary), residence (urban, rural), origin/homeland (highland, midland, lowland), and knowledge of malaria prevention methods. In this study, high land (“Dega”) is defined as the origin or homeland of seasonal migrant workers where malaria transmission is low or free of malaria, which is situated between 2000 and 2500 m above sea level; midland (“Woina Dega”) or highland fringes are geographic areas situated between 1500 and 2000 m above sea level and presented by both low and high malaria transmission, and lowland (“Kola”) is presented by altitudes less than 1500 m above sea level where malaria transmission is intense [37].

Ten data collectors were involved in the data collection from the departure. All data collectors have three to five years of experience in malaria data collection in the area. The quality of data collection was monitored daily by three supervisors and the principal investigator.

### Blood collection and microscopic blood examination

Blood collection was performed by each seasonal migrant worker who was interviewed. Ten data collectors who deployed for interviews were used for blood collection, and they had three to five years of experience in blood data collection for malaria. Both thick and thin blood smears were prepared from each selected seasonal migrant worker following standard operating procedures [38]. Two drops of blood were collected on a clean microscopic slide. One drop was used to prepare a thick smear, and the other was used to prepare a thin smear [39]. Finally, the slides were labeled with participant code and packed into a slide porter after being air-dried [40]. All slides were transported to Metema Hospital located in Genda Wuha town. The thin smear on each slide was fixed with absolute methanol, and both thick and thin smears were stained with 10% Giemsa for 10 min and examined microscopically under a light microscope for malaria parasites. Parasite results were reported based on screening of 100 microscopic fields at ×100 magnification. The initial thick film was classified as negative if no parasites were to be found after 500 white blood cells were counted. For quality assurance, 10% of positive slides were checked by a senior laboratory technician for species confirmation [41]. Accordingly, the conformation of species types and positive reports were checked, and there was no discrepancy between the first microscopists and the senior laboratory technician who controlled the quality.

### Variables of the study

Independent variables are socioeconomic, demographic, knowledge and practice of malaria and malaria prevention methods and environmental factors. The presence or absence of malaria parasites was a dependent variable.

### Data processing and analysis

Before entering the completed data, a database template was prepared using the software. Then, the quantitative data were entered into the database. Data quality was checked for completeness and consistency by running frequency and descriptive statistics.

After the quality check, descriptive statistics were carried out to determine the relative frequencies of all the survey variables using SPSS version 20. Appropriate graphs and tables were generated to show differences in the relative frequencies of various variables. Levels of association between various variables were determined by the Pearson X^2^ test in situations where the expected frequencies were less than five. Where appropriate, values and confidence intervals (CI) for odds ratios (OR) are shown. The data were fitted with bivariate and multiple logistic regression models to estimate the predictors of malaria transmission. Crude OR and adjusted OR were calculated. *P* values less than 0.05 were considered statistically significant.

### Ethical considerations

Ethical clearance was obtained from the institutional review board (IRB) of the University of Gondar. Then, the Ethical Committee of Amhara Regional Health Bureau (ARHB) was informed to obtain further permission. Local administrations were also informed of the permission and facilitation of the study. During data collection, informed consent was sought from all the study participants, and they were informed and assured that interviews and blood tests were completely voluntary. All data were confidential, and their names were linked to the data in any way. They were told that questions could be skipped or that the interview could be stopped if they felt uncomfortable at any point. Participants were not compensated for their participation, but those who were found to be positive for malaria parasites were given malaria treatment by a nearby health facility, based on the national malaria treatment guidelines. Care was taken not to link the collected information to the respondents by name. Data and information collected or analysed were kept confidential using code numbers for each completed questionnaire.

## Results

### Sociodemographic characteristics of seasonal migrant workers

A total of 1208 seasonal migrant workers were interviewed at departure sites from farm activities. Their mean age was 26.6 ± 5.4 years, and the median was 27 years (IQR = 8). The majority (99.4%) of the seasonal migrant farmworkers were male, and 646 (53.5%) were in the age range of 25–34. Of the study subjects, 479 (39.7%) were able to read and write, 716 (59.2%) were farmers and 772 (63.9%) were not married. Dominantly, 1183 (97.9%) were Amhara by ethnicity, and 1156 (95.7%) were Orthodox by religion (Table 1).

**Table 1:** Sociodemographic characteristics of seasonal farmworkers at departure phase in Metema district, Northwest Ethiopia, November 25-December 10, 2018 (n = 1208)

Variables	Frequency (%)
Sex	
Male	1201 (99.4)
Female	7 (0.6)
Age (in years)	
15–24	453 (37.5)
25–34	646 (53.5)
35 +	109 (9.0)
Education	
Illiterate	411 (34.0)
Read and write only	479 (39.7)
Elementary	110 (9.1)
Secondary and above	208 (17.2)
Occupation	
Farmer	716 (59.2)
Daily labourer	350 (29.0)
Student	142 (11.8)
Marital status	
Single	772 (63.9)
Married	397 (32.9)
Divorced	39 (3.2)
Religion	
Orthodox	1156 (95.7)
Muslim	38 (3.1)
Others	14 (1.2)
Ethnicity	
Amhara	1183 (97.9)
Others	25 (2.1)

### Prevalence of malaria

**Figure 2:**
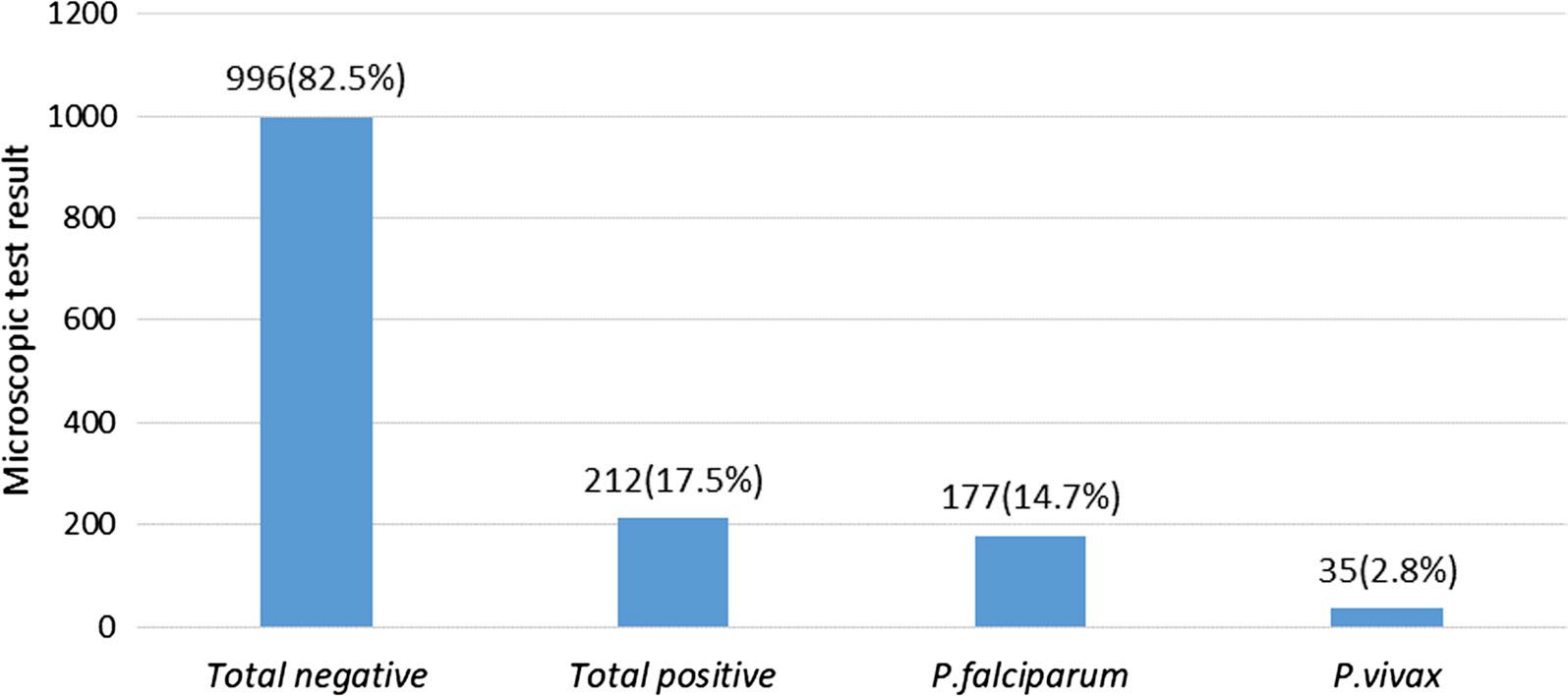
Prevalence of asymptomatic malaria at departure, northwest Ethiopia

The prevalence of malaria at departure was 17.5% (15.6–19.45%). The relative *Plasmodium* species of positive cases were 71.80% (177/212) *P. falciparum* and 28.20% (35/212) *P. vivax*. There was no mixed infection identified (Fig. 2).

### Travel history

The majority, 935 (77.4%), of the migrants were from rural residences. About 660 (54.6%) were from the highland, followed by 310 (25.7%) from the lowland (Table 2). Most 537 (44.5%) of the migrants visited one farm area, whereas 379 (31.4%), 229 (19.0%), and 63 (5.1%) of the study subjects visited two, three, and four and more farm sites during their stay for farm activities. At the departure phase, most of the migrants stayed outside the shelter from 6:00 PM up to midnight.

**Table 2:** Travel history of seasonal migrant workers at departure phase, Metema district, Northwest Ethiopia, November 25-December 10, 2018 (n = 1208)

Variables	N (%)	Asymptomatic malaria		X^2^	P-value
		Positive	Negative		
Residence					
Rural	935 (77.4)	175 (14.5%)	760 (62.9%)	3.893	0.048
Urban	273 (22.6)	37 (3.1%)	236 (19.5%)		
Origin/homeland					
High land	660 (54.6)	143 (11.8%)	517 (42.8%)	24.675	0.0001
Mid land	238 (19.7)	42 (3.5%)	196 (16.2%)		
Low land	310 (25.7)	27 (2.2%)	283 (23.4%)		
Time stayed outside the shelter					
6:00 PM–8:00 PM	250 (20.7%)	59 (4.9%)	191 (15.8%)	9.63	0.022
9:00PM–10:00PM	465 (38.5%)	67 (5.5%)	398 (32.9%)		
11:00PM–12:00PM	418 (34.6%)	74 (6.1%)	344 (28.5%)		
≥ 1:00AM	75 (6.2%)	12 (1.0%)	63 (5.2%)		
Number of farm sites visited					
One	537 (44.5%)	68 (5.6%)	469 (38.8%)	21.489	0.0001
Two	379 (31.4%)	70 (5.8%)	309 (25.6%)		
Three	229 (19.0%)	57 (4.7%)	172 (14.2%)		
Four and above	63 (5.1%)	17 (1.4%)	46 (3.8%)		
Days stayed at farm sites					
≤ 30	63 (5.2%)	6 (0.5%)	57 (4.7%)	322.8	0.0001
31–60	733 (60.7%)	100 (8.3%)	633 (52.6%)		
61–90	350 (28.9%)	88 (7.3%)	262 (21.5%)		
≤ 91	62 (5.2%)	18 (1.5%)	44 (3.7%)		

**Figure 3:**
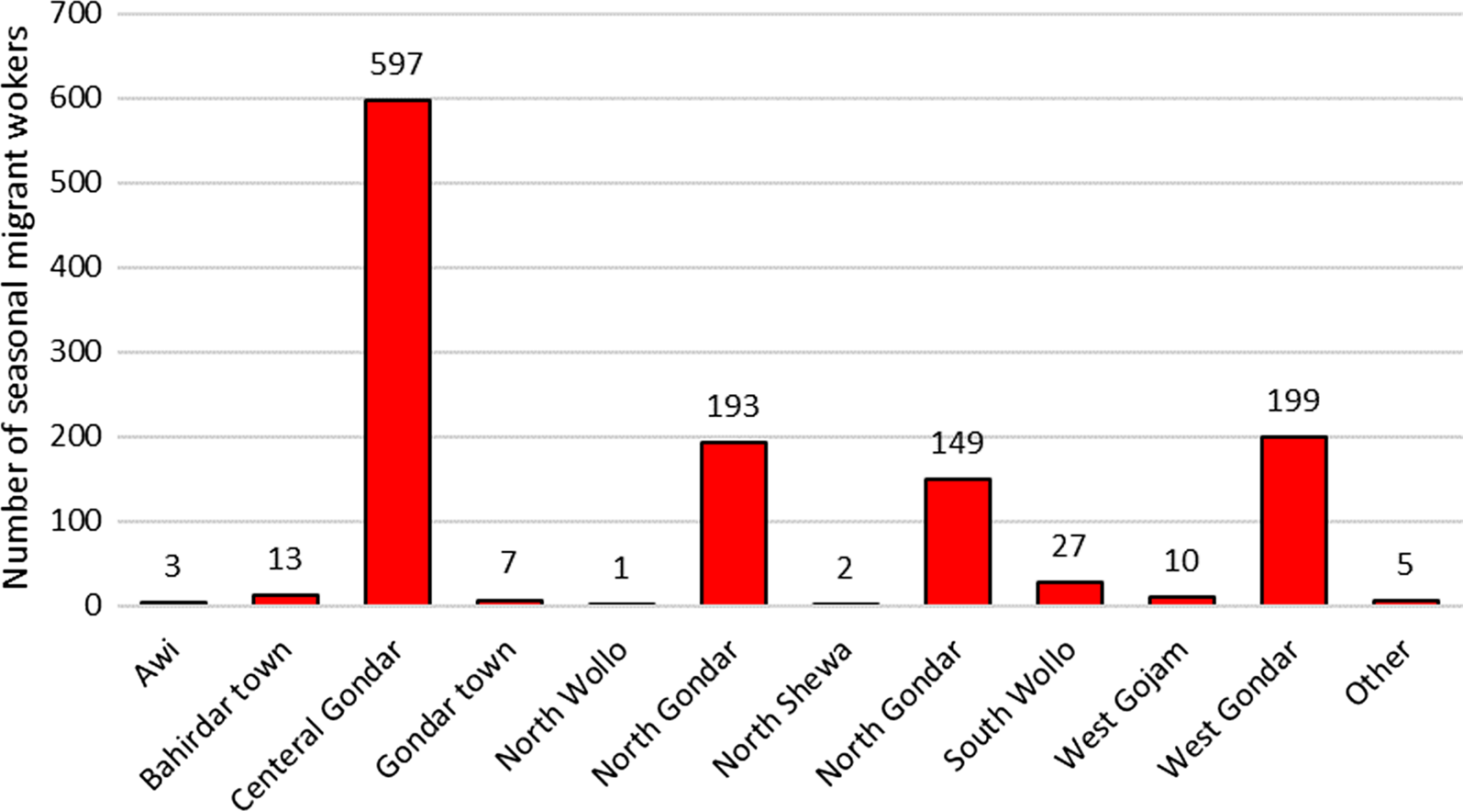
Number of seasonal migrant workers by their origin, northwest Ethiopia

**Figure 4:**
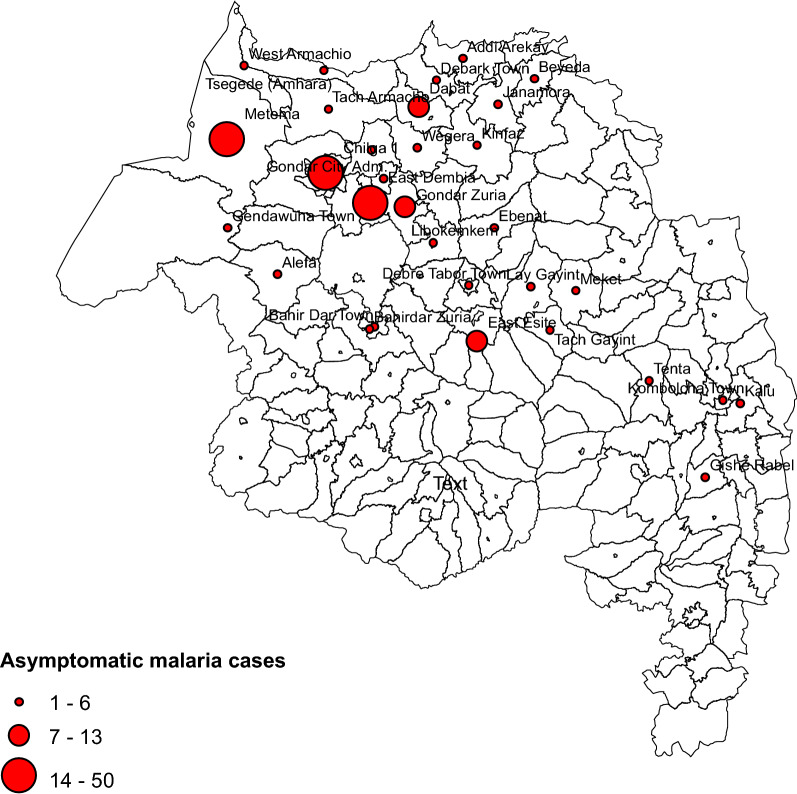
Asymptomatic malaria case distribution by origin/district, Northwest Ethiopia

Nearly all 1203 (99.6%) study subjects came from within the Amhara region and back to these areas (Fig. 3). Of these, 604 (49.4%) were from the Central Gondar zone, followed by West Gondar 199 (16.5%), North Gondar 193 (16.0%), and South Gondar 149 (12.3%). Approximately 771 (64%) of the study participants were from Dembia (23.4%) and Chilga (19.5%) woredas in the Central Gondar zone and Metema (14.6%) woreda in the West Gondar zone and Dabat woreda (6.4%) in the North Gondar zone. Asymptomatic malaria distribution by the district has shown that most of the seasonal migrant farmworkers upon return to Dembia (46), Chilga (42), and Metema (28) accounted for approximately 116 (54.7%) (Fig. 4).

The number of asymptomatic malaria cases was associated with the period stayed at farm sites (X^2^ = 322.8, P value = 0.0001). Majority of the seasonal migrant workers with asymptomatic malaria cases, 100 (8.3%), stayed at farm sites for 2 months, 31–60 days, and 88 (7.3%) of the cases stayed at farm sites for 3 months, 61–90 days (Table 2).

### The practice of malaria prevention methods

The practice of malaria prevention and control methods was assessed at departure. Accordingly, approximately 1054 (87.3%) of respondents had no LLINs, and only 154 (12.7%) of the seasonal migrants at departure had LLINs. Of 154 seasonal migrants, the majority 85 (55.2%) were using LLINs frequently, and 69 (44%) were occasionally using LLINs. LLIN use at departure was reported by 101 (65.6%) seasonal migrant workers on the last night. Very few 34 (2.8%) of the study subjects used repellents at the study site (Table 3).

**Table 3:** Practice of malaria prevention methods among seasonal migrant workers in Metema district, Northwest Ethiopia, November 25–December 10, 2018 (n = 1208)

Characteristics	Malaria parasites		Total (%)
	Positive (%)	Negative (%)	
Ownership of LLINs			
Yes	27 (2.2)	127 (10.5)	154 (12.7)
No	185 (15.3)	869 (72.1)	1054 (87.3)
Frequency of using LLINs			
Frequently	5 (3.2)	80 (51.9)	85 (55.2)
Occasionally	22 (14.3)	47 (30.5)	69 (44.8)
Use of LLINs in the last night			
Yes	10 (6.5)	91 (59.1)	101 (65.6)
No	17 (11.0)	36 (23.4)	53 (34.4)
Use of repellents			
Yes	6 (0.5)	28 (2.3)	34 (2.8)
No	206 (17.1)	968 (80.1)	1118 (97.2)
Wearing long sleeved clothes			
Yes	30 (2.5)	168 (13.9)	198 (16.4)
No	182 (15.1)	828 (68.5)	1010 (83.6)
Smoking			
Yes	9 (0.7)	66 (5.5)	75 (6.2)
No	203 (16.8)	930 (77.0)	1133 (93.7)

### Risk factors

Univariate analysis indicated that age, occupation, marital status, residence, origin/homeland, time to stay outside the shelter, number of farm sites visited and use of LLINs were significantly associated with malaria (Table 4).

**Table 4:** Predictors of asymptomatic malaria, Metema district, Northwest Ethiopia, November 25–December 10, 2018 (n = 1208)

Variables	Malaria parasites		COR	95% CI	AOR	95% CI
	Yes	No				
Age (in years)						
18–24	94	359	1		1	
25–34	100	546	0.57	0.42–0.79	0.55	0.38–0.80
35 +	18	91	0.49	0.27–0.90	0.65	0.32–1.30
Education						
No education	165	725	1			
Formal education	47	271	0.97	0.69–1.37		
Occupation						
Farmer	120	594	1		1	
Daily labourer	76	274	1.41	1.02–1.94	1.49	1.06–2.10
Student	16	128	0.88	0.53–1.45	0.74	0.43–1.25
Residence						
Rural	173	762	1		1	
Urban	39	234	0.68	0.46–0.99	0.75	0.50–1.12
Origin/homeland						
Highland	128	532	1		1	
Midland	47	262	0.77	0.53–1.13	0.96	0.64–1.44
Lowland	37	201	0.34	0.22–0.53	0.34	0.25–0.53
Number of farm sites visited						
One	57	480	1		1	
Two	77	302	1.56	1.08–2.25	1.58	1.08–2.32
Three	57	172	2.28	1.54–3.38	2.42	1.56–3.76
Four and above	21	42	2.55	1.38–4.69	3.16	1.64–6.11
Ownership of LLINs						
Yes	27	127	1			
No	185	869	1	0.64–1.56		
Utilization frequency of LLINs						
Frequently	5	80	1		1	
Occasionally	22	47	7.48	2.66–21.09	6.8	1.75–26.52
LLINs used in the last night						
Yes	10	91	1		1	
No	17	36	4.29	1.798–10.27	1.14	0.35–3.69
Repellent						
Yes	6	28	1			
No	206	968	0.99	0.41–1.24		
Wearing long-sleeved clothes						
Yes	30	168	1			
No	182	828	1.23	0.81–1.87		
Smoking						
Yes	9	66	1		1	
No	203	930	1.6	0.79–3.27	2.28	0.26–20.19

Multivariable analysis revealed that age, occupation, origin, number of farm sites being visited, and utilization of LLINs were significantly associated with the risk of malaria infection (*P* < *0.05*). The prevalence of malaria was not significantly associated with education, residence, ownership of LLINs, repellent, wearing long sleeved clothes, or smoking (*P* > *0.05*, Table 4).

Study subjects in the 25–34 age group (AOR = 0.551, 95% CI 0.378–0.804) were less likely to have malaria infection than those in the 18–24 age group. Similarly, the risk of malaria infection (AOR = 0.338, 95% CI 0.251–0.530) was lower among study subjects from low land areas than among study subjects from high land areas. Malaria prevalence was significantly higher among daily labourers (AOR = 1.497, 95% CI 1.065–2.105) than among farmers. On the other hand, seasonal migrant workers who had visited two farm areas (AOR = 1.588, 95% CI 1.085–2.324), three farm sites (AOR = 2.421, 95% CI 1.558–3.761), and four and more farm sites (AOR = 3.164, 95% CI 1.640–6.106) were significantly associated with malaria infection compared to migrants who visited only one farm site during their harvest time at the destination. The findings showed that as the number of visits to farm sites increased, the risk of malaria infection increased. The finding also revealed that irregular use of LLINs was statistically significant (AOR = 6.80, 95%CI 1.75–26.52) risk factor for asymptomatic malaria infection (Table 4).

## Discussion

This study characterized the role of seasonal migrant workers in carrying malaria with them while they were returning home. In this respect, asymptomatic malaria prevalence and risk factors were identified among seasonal migrant workers at the departure phase. Accordingly**,** asymptomatic malaria prevalence was identified, and multivariable logistic regression analysis revealed that age, occupation, origin, number of farm sites being visited, and utilization frequency of LLINs were significantly associated with asymptomatic malaria prevalence risk of malaria infection.

In this study, a significant amount of asymptomatic malaria infection was identified among seasonal migrant workers at departure. The results identified that the prevalence of asymptomatic malaria cases was 212 (17.5%) (Table 2), with a high proportion of *P. falciparum* 177 cases (71.8%). This was in line with the study conducted in West Armachiho district of Northwest Ethiopia and Dilla town in southern Ethiopia [42, 43] and India [44] and lower than the study conducted in East Shewa zone, Oromia, Ethiopia [45, 46], Nigeria [47], Tanzania [48], India [49] and China-Myanmar border, Southeast Asia [50] and higher than the study conducted in Gondar Zuria district of Northcentral Ethiopia and Democratic Republic of Congo [51, 52]. The possible reason for the high prevalence of asymptomatic malaria might be due to a significant proportion of seasonal migrant farmworkers who had repeated malaria exposure due to frequent visits to the farm areas in the previous year or those who came from malaria-endemic areas for harvesting that would facilitate the development of partial immunity and then carry the parasite for long periods without showing clinical signs and symptoms [53–55]. Asymptomatic malaria cases might be responsible for spreading malaria in areas where they are passing through while they are returning home and their communities. A study conducted in villages around Lake Tana, Northwest Ethiopia, indicated that travel to farms in the lowlands was significantly associated with the risk of malaria infection and imported malaria (91.5%) to the villages [8]. Moreover, the possible reasons for the difference could be differences in study design, geographical location, nature of study population, sample size, tool used, study period, and the implemented malaria control program in the study area.

In this study, age was considered one of the most important factors associated with asymptomatic malaria infection at departure [56]. The age group from 25 to 34 years of age was less likely to have asymptomatic malaria infection compared with the age group from 18 to 24 years of age. This was in agreement with the studies conducted in Ethiopia [52] and Yemen, where adults were predominantly asymptomatic malaria carriers compared to children [57]. The reference age group is more at risk of malaria infection than adults who are asymptomatic parasite carriers because they have acquired strong immunity from repeated exposures to the malaria parasite. Moreover, high exposure in farm areas, visiting various farm sites, and less use of preventive methods put this age group more at risk of malaria in the study area.

In this study, occupation was significantly associated with a high risk of malaria infection. Being daily labourers (individuals who work for daily wages) was at increased risk of asymptomatic malaria infection compared to farmers (persons whose farming is the main source of income). This might be due to high exposure to malaria infection that might be related to low income [58], less access to malaria information [8] and behaviour [42], less access to malaria prevention methods [25, 59], and having no access to health care [25]. According to a study conducted in Dembia district, Northcentral Ethiopia, low malaria information was responsible for high malaria prevalence among study subjects who had a travel history to low land malarial areas [8]. However, there was no significant association of malaria prevalence among students.

This study revealed that most of the seasonal migrant farmworkers were from rural residences in high land areas and low land areas. The risk of malaria infection is high among migrants from highland areas due to low immunity to malaria. This might be the reason for the high malaria prevalence in this study group at departure. This was in agreement with the study conducted in West Armachiho district, Northwest Ethiopia [42]. There was evidence that travellers from high land areas to low land areas for farm activities were responsible for the spread of malaria to high land areas [54].

In this study, the number of farm sites being visited by study subjects was associated with the risk of asymptomatic malaria at departure. It was found that as the number of farm sites being visited increased, the risk of asymptomatic malaria increased (Table 4). Seasonal migrant workers who had visited two, three, and four and above were significantly associated with the prevalence of asymptomatic malaria compared to seasonal migrant workers who had visited one farm site during their harvest time at development corridors. Visits of three and four farm sites were more than two and three times at risk of asymptomatic malaria infection compared to having one farm site visit.

In the current study, the ownership of LLINs was low among the study subjects. Only 12.7% of them possessed LLINs. LLIN ownership was lower than that in other studies in the area, showing 32.4% in 2014 [23], 64% in 2015 national MIS [60], and 31% in 2016 [61]. Moreover, the ownership was also lower than the study findings from Cambodia [62]. Of the 154 (12.7%) seasonal migrant workers who owned LLINs, 85 (55.2%) were using LLINs daily. LLIN utilization was similar to that in a study conducted among seasonal migrant workers in Myanmar [63]. However, the rate was higher than that in a study conducted in Ethiopia, which was 29% in 2019 [64]. The study findings also revealed that approximately 66% of those who owned LLINs slept under LLINs the previous night. This finding was higher than that of a study conducted in Myanmar showing 50% among seasonal migrants [63]. This might be associated with low access to LLINs, and most of the seasonal migrant workers did not bring their LLINs from home to farm areas.

This study kept its strength by taking increased samples and sampling techniques to minimize selection bias and to ensure internal and external validity. Confounding factors were also included in the binary logistic regression analysis. Asymptomatic malaria prevalence and associated risk factors were investigated to determine the role of seasonal migrant workers in transporting malaria parasites to their origin upon return and the risk of spreading and challenging malaria prevention and control activities as well as elimination goals in the country. Failing to use the molecular tool PCR in detecting asymptomatic malaria to support microscopic investigation was a limitation of this study.

## Conclusion

In this study, asymptomatic malaria remains high among seasonal migrant workers departing home from malaria-endemic areas. This may fuel a resurgence of malaria transmission in the high lands and cause challenges to country’s malaria control and elimination efforts. Frequent visits to farm sites and occasional use of LLINs were the major risk factors that might expose migrants to increased asymptomatic malaria infection. Expansion of malaria treatment services (outreach) in the farm areas and deployment of adequate health workers could provide access to migrant workers who visit different farm sites frequently [25, 65]. Moreover, asymptomatic malaria tests and treatment of positive cases at departure and transit could help to reduce the spread of malaria at origin [41]; there should be integration between malaria officers at migrants’ origin and departure for further follow-up to decrease any risk of spread and conduct further study to understand their malaria contribution and to integrate that with local malaria prevention and elimination.

## Data Availability

Data and all the necessary materials are available with the corresponding author upon request.

## Abbreviations

ACT: Artemisinin-based combination therapy
AOR: Adjusted Odds Ration
ARHB: Amhara Regional Health Bureau
CI: Confidence Interval
IRS: Indoor Residual Spraying
IRB: Institutional Review Board
IQR: Interquartile range
LLINs: Long-lasting insecticidal nets
PCR: Polymerase chain reaction
RDT: Rapid diagnostic test
SPSS: Statistical Package for the Social Sciences
WHO: World Health Organization
